# 119. Performance of Infectious Diseases Specialists, Hospitalists, and Generalists in Case-Based Scenarios Illustrating Antimicrobial Stewardship Principles at 16 VA Medical Centers

**DOI:** 10.1093/ofid/ofab466.321

**Published:** 2021-12-04

**Authors:** Christopher J Graber, Alissa Simon, Yue Zhang, Matthew B Goetz, Matthew B Goetz, Makoto M Jones, Jorie M Butler, Ann F Chou, Peter A Glassman

**Affiliations:** 1 VA Greater Los Angeles Healthcare System/UCLA, Los Angeles, California; 2 VA Greater Los Angeles Healthcare System, Los Angeles, California; 3 University of Utah, Salt Lake City, UT; 4 VA Greater Los Angeles Healthcare System and David Geffen School of Medicine at UCLA, VA-CDC Practice-Based Research Network, Los Angeles, California; 5 Salt Lake City VA/University of Utah, Salt Lake City, Utah; 6 Oklahoma University Health Sciences Center, Oklahoma City, Oklahoma

## Abstract

**Background:**

As part of a project to implement and evaluate antimicrobial dashboards at selected VA facilities nationwide, we assessed provider attitudes and knowledge related to antibiotic prescribing among physicians working in inpatient settings at 16 VA facilities.

**Methods:**

The online survey explored attitudes toward antimicrobial use and assessed respondents’ management of four clinical scenarios: cellulitis, community-acquired pneumonia (CAP), non-catheter-associated asymptomatic bacteriuria (NC-ASB), and catheter-associated asymptomatic bacteriuria (C-ASB). Responses were scored by assigning +1 for an answer most consistent with guidelines, 0 for a less-guideline-concordant but acceptable answer and -1 for an incorrect answer. Scores were normalized to 100% correct to 100% incorrect across all questions within a scenario, and mean scores were calculated across respondents by specialty; differences in mean score per scenario were tested using ANOVA.

**Results:**

One-hundred-thirty-nine physicians completed the survey (n=19 ID physicians, 62 hospitalists, 58 generalists). Attitudes were similar across the three specialties. There was a significant difference in cellulitis scenario scores (correct responses: ID=67.4%, hospitalists=51.2%, generalists=41.8% correct, p=0.0087). Scores were not significantly different across specialties for CAP (correct responses: ID 76.2%, hospitalists 63%, generalists 56.5%, p=0.0914) and NC-ASB (correct responses; ID 63%, hospitalists 55%, generalists 36.2%, p=0.322), though ID trended higher. Lowest scores were observed for C-ASB (ID 39.5% correct, hospitalists 4% incorrect, generalists 8.5% incorrect, p=0.12).

**Conclusion:**

Significant differences in performance on management of cellulitis and low overall scores on C-ASB management point to these conditions as being potentially high-yield targets for antimicrobial stewardship interventions.

**Disclosures:**

**Matthew B. Goetz, MD**, Nothing to disclose **Peter A. Glassman, MBBS**, **US Pharmacopeia (formerly), PAG; Kaiser Permanente (current employee, spouse**) (Advisor or Review Panel member, The above refers to USP (ended in 2020).)

